# HIV-1 Group M Conserved Elements Vaccine

**DOI:** 10.1371/journal.ppat.0030157

**Published:** 2007-11-30

**Authors:** Morgane Rolland, David C Nickle, James I Mullins

**Affiliations:** The Scripps Research Institute, United States of America

Recent HIV vaccine designs have sought to block viral escape pathways by compressing antigenic diversity. In light of HIV's propensity to mutate and thereby to ever ramify viral populations, could it be that providing sufficient protection against global diversity is an insurmountable problem? We propose an alternative HIV-1 vaccine design that deliberately includes viral segments conserved across the entire main group (or M group) of HIV-1 strains and excludes variable segments. We describe a prototype conserved elements (CE) vaccine constituted of 45 viral segments at least eight amino acids long that fulfill stringent conservation criteria.

Our paradigm contends that the best way to cope with HIV-1 diversity may be to avoid it altogether. We argue that a successful vaccine must elicit responses against conserved regions of the viral proteome in which mutations would severely compromise the viability of the virus. Simultaneously, it must not elicit responses against variable, “decoy” elements of the virus, i.e., features that can mutate while retaining function, and that can absorb much of the adaptive host immune response.

Coping with HIV-1′s extensive diversity is a major challenge for vaccine design strategies. Centralized (consensus and ancestral) immunogens [1–3] have in some cases improved the breadth of responses, and recent designs seek to compress the more common variant features among circulating strains into immunogens [4–6]. However, there is a practical limit to antigenic complexity that may prevent inclusion of all escape pathways in realistically sized immunogens. Besides, HIV's propensity to mutate has been shown to provide means for HIV to escape from antiretrovirals and antibody and cytotoxic T lymphocyte (CTL) pressures.

The foregoing considerations led us to propose a vaccine exclusively composed of viral segments strictly conserved in all HIV-1 M group proteins and specifically devoid of mutable segments. The presence of segments that are nearly invariant in all HIV-1 M group proteomes strongly suggests that those CE are both obligatory for viral viability and are the Achilles' heels of the virus. Additionally, considering that variable segments can readily escape CTL pressures and can be highly immunogenic epitopes, we propose that mutable segments may act as immunologic decoys, subverting responses away from conserved elements.

## Rationale

**Figure 1:**
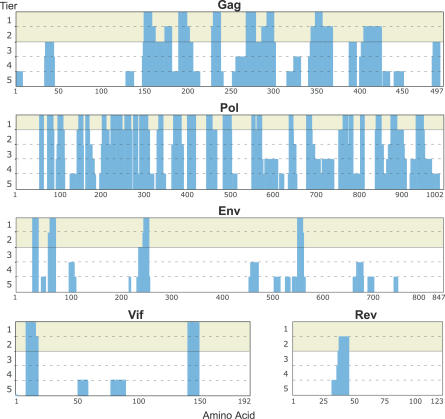
Conservation of M Group Sequences across HIV-1 Proteins The full lengths of each of the viral proteins containing 1st and/or 2nd tier CE are shown. The histogram indicates the tier of conservation for each segment of at least eight AAs in length, with tier number shown on the *y*-axis. 1st tier segments correspond to segments at least eight AAs long in which the AA at each site is found in more than 98% of HIVDB database sequences. 2nd tier include sites at which the most common AA is found in less than 98% of sequences in the database, but at which two AAs together make up more than 99% of the AAs found at that site in the database. For comparison, additional tiers of potential use for vaccine design are shown. 3rd tier expands the set to include two variable sites, and 4th tier includes *n* variable sites, each of which satisfy the criteria of having two AAs that together make up more than 99% of the AAs in the database. The 5th tier corresponds to peptides that have *n* variable sites that satisfy the criteria for 4th tier, but the requirement for conservation encompassed by the two AAs at each site is relaxed to more than 98%.

Despite HIV-1′s extreme diversity, certain segments are nearly invariant (Figure 1). Near-total conservation of some sites implies that tight functional constraints obviate certain mutations. Indeed, escape from CTL responses, as with antiretroviral resistance, sometimes results in fitness decrease [7–14]. Specifically, escape mutations in Gag impaired viral fitness significantly more than mutations in Env (*p* = 0.0033, Mann-Whitney test) (unpublished data). These findings corroborate the amount of polymorphisms that can typically be accommodated in each protein, as also hinted by a study of *env* V3 arguing for site-specific amino acid (AA) conservation as a predictor of viral fitness [15].

As HIV-1 establishes infection, it relentlessly mutates away from the founding strain [16]. However, some mutations recover consensus-like AAs [14,17,18], and these transitions to ancestral states may reverse CTL escape mutations back to susceptible and possibly increasingly fit forms upon transmission to human leukocyte antigen (HLA)-mismatched individuals [10,11,18,19,20].

HIV-1′s predilection to mutate away from CTL-susceptible sequences highlights the critical importance of CTL responses. Gag, the second-most conserved HIV-1 protein, is immunodominant during infection [21] in different ethnic groups [22]. Numerous studies have suggested a superior role for Gag-specific CTL in viral containment [23–33], whereas CTL targeting of variable proteins may not contribute or even negatively impact immune control in HIV infection in humans [23,30] and in mouse studies [34]. Likewise, the emergence of escapes from Env-specific antibodies against variable regions hinders efforts to generate neutralizing antibodies [35,36].

Many studies underscored the challenge of broadening CTL recognition through vaccination as they reflected typical HIV immunodominance profiles: the immune system focuses on relatively few immunodominant epitopes, leaving many epitopes subdominant or cryptic. Since subdominant responses may be critical to effective suppression [37], mitigating immunodominance patterns could prove critical for successful vaccines.

Those considerations converge onto the approach of exclusively using conserved regions as components of HIV-1 vaccines [38–40]. Focusing on CTL induction, we articulate the conserved elements approach to vaccine design and describe here our candidate antigen. We consider that: (1) an HIV vaccine must be composed of rigorously conserved elements of the virus that cannot mutate without greatly deterring or eliminating viability; (2) mutable epitopes can act as immunodominant decoys, thereby sapping responses against protective, invariant epitopes; and (3) most HIV targets that can mutate without drastically impairing virus functionality do not contribute to the durable efficacy of the immune response.

## CE Vaccine Design

To define CE, we evaluated the conservation in a database of all HIV-1 M group nucleotide sequences available (one sequence/person; sequence accession numbers and alignments available at http://mullinslab.microbiol.washington.edu/HIV/Rolland2007/). We then identified protein stretches at least eight AAs long, the minimal size of a CTL epitope, in which every AA is nearly conserved across the entire HIV-1 M group. Given the preponderance of defective HIV sequences in vivo, we did not require absolute conservation but included segments where each AA is found in more than 98% of all HIV-1 M group sequences (1st tier CE). This level of conservation is consistent with our recent study of fitness loss associated with mutations in Gag (unpublished observations). We have also included CE in which two variant forms at a given position together account for more than 99% of the amino acids found in the database (2nd tier CE) (Figure 1). Although the Los Alamos HIV sequence database (HIVDB) is overwhelmingly constituted of clades B and C, other M group subtypes were sufficiently represented in sequence alignments such that a subtype-specific AA would drag that site below the 1st tier criterion of conservation.

Most 1st and 2nd tier CE were identified in Pol (*n* = 27; only 1st tier peptides were included in our CE, due to the extreme conservation of Pol) and Gag (*n* = 10), the two most conserved HIV-1 proteins; however, other CE were found in Env (five), Vif (two) and Rev (one) (Table 1). The idiosyncrasy of the CE design means that more than half of CE peptides correspond to Pol segments. However, it should be noted that Pol epitopes are rarely targeted in natural infection. Recent results suggest that the infrequent recognition of Pol epitopes is likely related to the considerably lower ratio of Pol compared to Gag proteins produced by the virus [41] rather than to a lack of immunogenicity. Both observations raise concerns about Pol segments as sagacious choices for vaccine candidates.

**Table 1:**
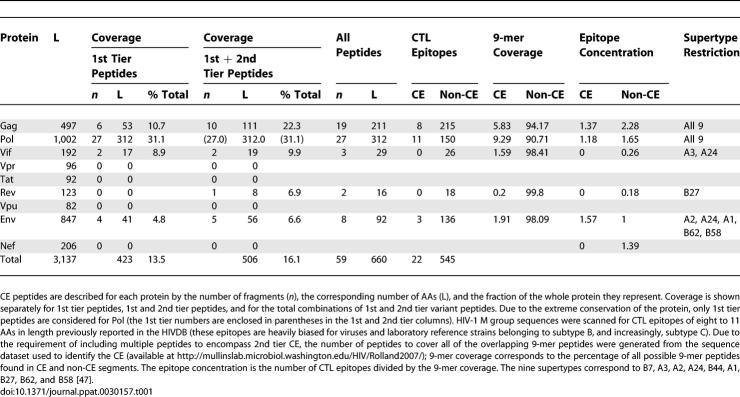
CE Peptide Coverage

Indeed, early vaccine trial results show a preponderance of CTL responses in Pol when Gag and Pol are administered at the same ratio: a study of immune responses elicited by an Ad5 vaccine candidate in 30 HIV-uninfected participants showed that 39 distinct epitopes were recognized in Pol and eight in Gag (Helen Horton, personal communication). As another consequence of not defining our candidate vaccine based on the frequency of recognition in natural infection, our CE vaccine design does not contain Nef epitopes, a traditional component of HIV-1 vaccine candidates, including the recently failed Merck/STEP clinical trial. We reason that while both Gag and Nef are under important CTL pressure, the degree of polymorphisms that can be accommodated in Nef leads to a cycle of CTL escape mutations/de novo responses to variant epitopes, and those readily escapable shifting CTL responses do not afford long-term control of HIV-1 replication. In contrast, a CTL escape mutation in Gag has a strong impact on viral viability and those strong constraints on HIV-1 evolution might coerce the virus toward minimal replicative fitness capacities. Likewise, we consider that a CTL-based vaccine would not benefit from focusing largely on Env antigens.

The CE segments contain known CTL epitopes, including some previously associated with long-term nonprogression or recurrent HIV exposure without seroconversion [42–46] (Note: CE sequences are available at http://mullinslab.microbiol.washington.edu/HIV/Rolland2007/). Some epitopes are strictly embedded in CE: eight in Gag, 11 in Pol, and three in Env, representing a variety of HLA restrictions. In addition, CE sequences include supertype motifs from all nine supertypes (B7, A3, A2, A24, B44, A1, B27, B62, B58) in both Gag and Pol, providing a population coverage of at least 80%, regardless of ethnicity [47]. Additional epitopes were found to overlap CE/non-CE junctions (data not shown); thus extending CE immunogens could increase the number of peptides available to CD8^+^ T lymphocytes. However, their inclusion would relax our stringent conservation criteria. It should be noted that attempts to correlate CTL breadth and viral containment have been inconclusive [25,26,48–51]. The conundrum of extending CE without including potentially pathogenic variable positions thus begs for a better understanding of virus viability with respect to sequence conservation.

One intricate issue with the CE strategy is to ensure that antigen structures are recognized outside of native proteins. Thus, CE constructs have to be engineered optimally to elicit immune responses by capitalizing on the mechanisms governing epitope processing and presentation while preventing the creation of junctional immunogenicity or homology to the HIV or human proteome [52]. Furthermore, certain elements of the HIV proteome may be conserved because there is a dearth of features within and surrounding these elements that are capable of mediating efficient processing for presentation on HLA. Such elements would not be useful for inclusion in a vaccine. Thus, configurations should explicitly capitalize on emerging data on the mechanisms that govern epitope processing and presentation.

In eliciting responses to particular segments, another undefined problem is immunodominance [22,53]. With respect to all 9-mer peptides in datasets, epitope concentrations were lower in CE than in non-CE, except in Env (Table 1). However, epitopes in variable regions like Env are under-represented in databases [18] and HLA class I allele promiscuity is more pervasive than previously thought [54]. In terms of the utility of a CE vaccine, considering the lower representation of epitopes in CE versus non-CE as problematic may be misleading, especially as our CE design is intended to not reproduce certain features of the antiviral response seen in natural infection. Since CE are apparently not preferentially targeted in natural infection, CE vaccine designs have to deliberately focus the immune response toward segments that are not traditionally immunodominant, thereby assuring that reactivity against CE is not obscured by the immunodominating responses evident in natural infection. For example, isolating subdominant epitopes in CE vaccines might promote their recognition [55,56].

## Concluding Remarks

To overcome the stumbling block posed by HIV-1 diversity, we specified a novel HIV vaccine design that exclusively includes portions of the proteome meeting stringent conservation criteria and thereby explicitly deprived of mutable segments. We consider that variable regions act as decoys that divert the immune system from responding to conserved regions, which are seemingly better for long-term viral control. Crucially, our scheme culminates in a single global CE vaccine suited for evaluation against all circulating HIV-1 M group strains. 

## Abbreviations

AA: amino acid
CE: conserved elements
CTL: cytotoxic T lymphocyte
HIVDB: HIV sequence database
HLA: human leukocyte antigen
M: main
